# Characterising the compressive anisotropic properties of analogue bone using optical strain measurement

**DOI:** 10.1177/0954411919855150

**Published:** 2019-06-18

**Authors:** Alex D Marter, Alexander S Dickinson, Fabrice Pierron, Yin Ki (Kiki) Fong, Martin Browne

**Affiliations:** 1 Bioengineering Science Research Group, Faculty of Engineering and Physical Sciences, University of Southampton, Southampton, UK; 2 Engineering Materials Research Group, Faculty of Engineering and Physical Sciences, University of Southampton, Southampton, UK

**Keywords:** Analogue bone, polyurethane foam, material characterisation, mechanical testing, compression testing

## Abstract

The validity of conclusions drawn from pre-clinical tests on orthopaedic devices depends upon accurate characterisation of the support materials: frequently, polymer foam analogues. These materials often display anisotropic mechanical behaviour, which may considerably influence computational modelling predictions and interpretation of experiments. Therefore, this study sought to characterise the anisotropic mechanical properties of a range of commonly used analogue bone materials, using non-contact multi-point optical extensometry method to account for the effects of machine compliance and uneven loading. Testing was conducted on commercially available ‘cellular’, ‘solid’ and ‘open-cell’ Sawbone blocks with a range of densities. Solid foams behaved largely isotropically. However, across the available density range of cellular foams, the average Young’s modulus was 23%–31% lower (p < 0.005) perpendicular to the foaming direction than parallel to it, indicating elongation of cells with foaming. The average Young’s modulus of open-celled foams was 25%–59% higher (p < 0.05) perpendicular to the foaming direction than parallel to it. This is thought to result from solid planes of material that were observed perpendicular to the foaming direction, stiffening the bulk material. The presented data represent a reference to help researchers design, model and interpret tests using these materials.

## Introduction

Polymer foams have been extensively used in the testing and development of orthopaedic devices and corresponding computational models.^1–5^ Often these foams are used in preference to cadaver and animal material, with researchers noting their relative low cost, availability, the consistency of material properties, avoidance of ethical concerns and their ease of handling and storage.^6^ A range of polymer foam types is available commercially (Sawbones; Pacific Research Labs, Malmö, Sweden), as both anatomically shaped bone models and standard blocks, to represent a range of bone types. The mechanical properties of polymer foams may be adjusted by means of porosity content, to cover a range of natural bone stiffness. However, the polymer expands by ‘foaming’ during manufacture, which may result in an uneven aspect ratio of the foam structural features (i.e. cells), and consequent anisotropic mechanical behaviour and will lead to varying mechanical behaviour dependent on the orientation of testing.^7,8^

A number of studies have evaluated the mechanical properties of polyurethane (PU) foams in the context of a biological analogue, considering compressive,^9–13^ shear^13^ and fatigue^14^ properties. However, to the authors’ knowledge, their anisotropic material properties have not been reported and may be of key importance to computational models and analogue material selection. In addition, limited Poisson’s ratio data are available for PU foams commonly used as a biological analogue. In this study, the assumption of isotropy was tested both parallel and perpendicular to the foaming direction. Literature data indicate a wide range of Young’s modulus values for nominally the same material,^11–13,15,16^ which is highly dependent upon the experimental method employed. Therefore, testing was performed using a non-contact multi-point optical extensometry method that accounts for the effects of machine compliance and uneven loading. This method has previously been verified against the digital volume correlation method.^15,16^

## Method

**Figure 1.**
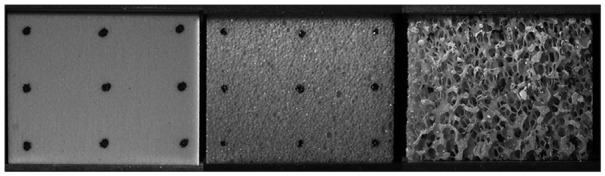
Representative sample images for solid polyurethane (S, left), cellular polyurethane (C, centre) and open urethane–epoxy composite (O, right) foam types.

A range of sample types and densities was selected corresponding to a range of trabecular bone material properties spanning the majority of commercially available materials. Three different types of foam were tested (Figure 1): ‘solid rigid polyurethane’ (S), ‘cellular rigid polyurethane’ (C) and ‘open cell rigid foam’ (O, a composite made of urethanes, epoxies and structural fillers; Sawbones^®^, Malmö, Sweden). Where available, densities of each foam type were selected such that they were directly comparable between foam types (Table 1). All specimens were stored and tested in ambient environmental conditions.

**Table 1.** Manufacturer-quoted test material properties and test finish load.

Foam type	Manufacturerproduct code	Quoted density		Yield stress (MPa)	Maximum test load(N; parallel to foaming direction)	Maximum testload (N; perpendicularto foaming direction)
		(pcf)	(g/cm^3^)			
Cellular	1522-09	7.5	0.120	1.4	1820	1430
	1522-11	12.5	0.200	3.9	5070	3980
	1522-1300	15	0.240	4.1	5330	4180
	1522-12^a^	20	0.320	5.4	7020	5510
Solid	1522-536	8	0.128	1.5	1950	1530
	1522-48	12	0.192	3.2	4160	3260
	1522-02	15	0.240	4.9	6370	5000
	1522-03	20	0.320	8.4	10,920	8570
	1522-04	30	0.480	18	23,410	18,360
Open	1522-524	15	0.240	0.67	870	680
	1522-526-1	20	0.320	1.3	1640	1280
	1522-525	30	0.480	3.20	4160	3260

a The 20 pcf (0.306 g/cm^3^) cellular foam has glass fibre reinforcement.

Samples were cut to 40 × 51 × 51 mm (nominal dimensions) using a bandsaw, to adhere to testing standard ASTM D1621 – 10 in the foaming direction. The foaming direction was identified as the smallest 40 mm ‘thickness’ dimension of the blocks as supplied by the manufacturer. Six specimens were tested for each foam type and density. The apparent density of each specimen was calculated by measurement of dimensions by digital callipers and mass by electronic balance, with precisions of 0.01 mm and 0.0001 g, respectively, in accordance with ASTM D1622 and compared with the manufacturer-quoted densities.

**Figure 2.**
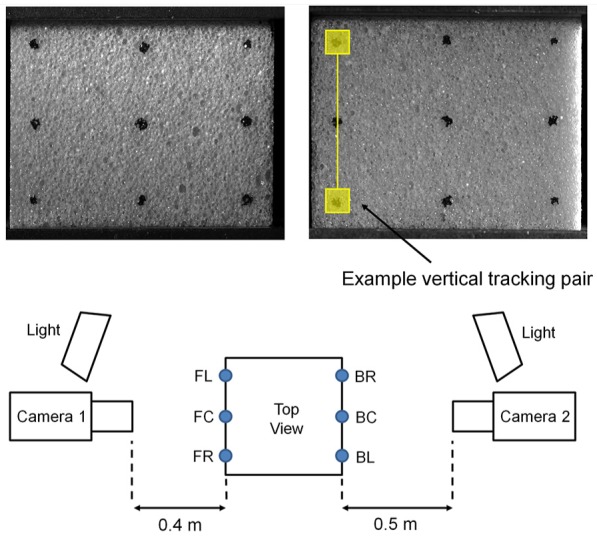
Marker arrangement and experimental schematic for optical extensometry.

Each sample was compressed in a screw-driven electromechanical testing machine (Instron 5569; Instron, High Wycombe, UK). A displacement rate of 0.5 mm/min was selected to minimise test duration and limit image motion throughout testing. To maintain testing within the material’s elastic behaviour range and thus enable testing of the same specimen in two directions, each specimen was loaded to half its documented yield stress (Table 1). Specimen deformation was measured by a non-contact optical extensometry method as described by Marter and colleagues.^15,16^ A grid of nine markers was drawn onto the front and back surfaces of each specimen (Figure 2). These markers were then recorded throughout loading using two cameras (AVT Manta G-504B, 2452 × 2056 pixels, 8-bit) fitted with a fixed focal length lens (Sigma 105 mm f/2.8 EX DG Macro). Image exposure time was set to 1000 µs to minimise motion blur while maintaining image contrast. A laser cut acrylic template was used to ensure repeatability of point marker locations on the specimen’s surface. The heterogeneous surface of the open-cell samples complicated this marking procedure. Where markers could not be placed on the specimen surface, the material’s surface structure was used to provide trackable features. The averaged strain response of the six vertical marker pairs was used to calculate specimen Young’s modulus. Poisson’s ratio of each specimen was calculated as the ratio of the averaged central horizontal marker pair’s strain response divided by the averaged vertical strain response. The central horizontal marker pairs were used to minimise the influence of friction at the specimen ends. Both Young’s modulus and Poisson’s ratio results were corrected to account for differences in surface to volumetric strains, not captured by point tracking, using an ANSYS finite element model.^15,16^

Anisotropy of each specimen was assessed by testing both along (parallel to) and perpendicular to the foaming direction, with the assumption that the material was transversely isotropic. As specimens were not cubic, maximum test loads for each loading direction were adjusted such that the final stress was equal for all tests (Table 1). Young’s modulus and Poisson’s ratio results were tested for normality using the Shapiro–Wilk test. For normally distributed data, a paired, two-tailed t-test was used to test the null hypothesis that the materials had the same Young’s modulus or Poisson’s ratio parallel and perpendicular to the foaming direction, with a 95% significance level. For non-parametric data, a Wilcoxon signed-rank test was used.

## Results

Cellular and solid foams had consistent densities (Table 2), with the standard deviation of density being small compared to the averaged measured value for each sample group (coefficient of variation <2%). The open-cell foams were more heterogeneous (coefficient of variation 4%–8%).

**Table 2.** Measured sample densities.

Foam type	Quoted density(g/cm^3^)	Measured densityMean (SD; g/cm^3^)
Cellular	0.120	0.115 (0.001)
	0.200	0.206 (0.001)
	0.240	0.248 (0.001)
	0.320	0.306 (0.001)
Solid	0.128	0.124 (0.001)
	0.192	0.183 (0.004)
	0.240	0.240 (0.000)
	0.320	0.311 (0.001)
	0.480	0.455 (0.004)
Open	0.240	0.239 (0.020)
	0.320	0.329 (0.012)
	0.480	0.461 (0.022)

### Foaming direction

**Figure 3.**
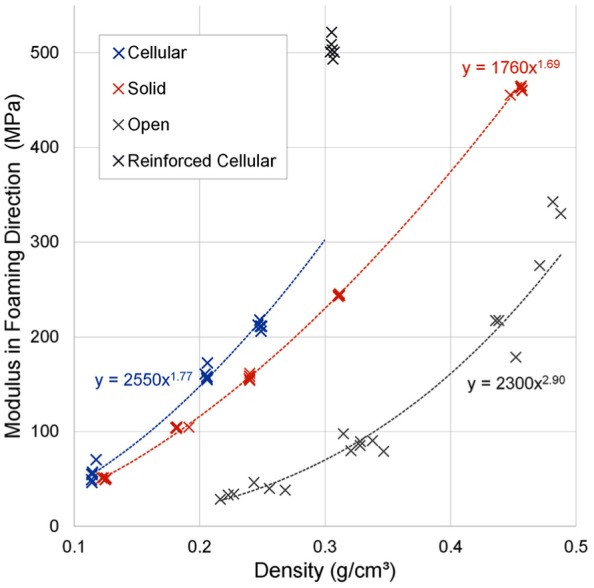
Summary of all point tracking results for tested foam types and densities compressed in the foaming direction. 0.306 g/cm^3^ density cellular foam excluded from power law regression due to the inclusion of fibre reinforcement.

Foams showed power law relationships (R^2^ > 0.97) between the measured Young’s modulus and density, for all foam types when tested in the foaming direction (Figure 3). The cellular foam followed a similar trend at lower densities, but the 0.306 g/cm^3^ density foam was an outlier, owing to its E-glass reinforcement, which was not present in the solid PU foam, or the other grades of cellular foam.

Using these power laws and adjusting where necessary for equal material densities, the cellular foams had 22%–29% higher average Young’s modulus than solid foams, with the exception of the fibre-reinforced cellular 0.306 g/cm^3^ density foam, which was 107% higher (Figure 3). The open-cell foams had considerably lower modulus on average than other foam types of same densities (23%–54% that of solid foams). All differences were significant (p < 0.005; Table 3).

**Table 3.** Summary of Young’s modulus and Poisson’s ratio values calculated from each testing directions

Foam type	Nom. density		Young’s modulus results			Poisson’s ratio results		
			Mean (SD), MPa			Mean (SD)		
	(pcf)	(kg/m^3^)	Foaming	Transverse	Significance	Foaming	Transverse	Significance
Cellular	7.5	115	55.5 (8.27)	38.8 (2.95)	0.002*	0.41 (0.077)	0.33 (0.020)	0.033*
	12.5	206	160 (6.40)	120 (3.60)	0.028* ^a^	0.39 (0.023)	0.31 (0.008)	<0.001*
	15	248	212 (3.81)	164 (2.49)	<0.001*	0.34 (0.023)	0.31 (0.011)	0.033*
	20^b^	306	505 (9.70)	357 (17.9)	<0.001*	0.33 (0.021)	0.28 (0.054)	0.028* ^a^
Solid	8	124	50.5 (0.83)	52.6 (4.06)	0.231	0.31 (0.003)	0.34 (0.033)	0.345^a^
	12	183	104 (0.68)	103 (2.50)	0.180	0.34 (0.005)	0.31 (0.013)	0.004*
	15	240	157 (2.83)	155 (3.08)	0.193	0.32 (0.005)	0.30 (0.007)	0.001*
	20	311	244 (0.93)	249 (9.10)	0.206	0.31 (0.003)	0.32 (0.009)	0.083
	30	455	461 (3.55)	457 (8.66)	0.128	0.32 (0.002)	0.31 (0.010)	0.055
Open	15	239	36.8 (6.14)	58.5 (18.5)	0.022*	0.47 (0.067)	0.23 (0.074)	0.002*
	20	329	87.0 (7.07)	138 (26.0)	0.006*	0.31 (0.052)	0.24 (0.057)	0.051
	30	461	260 (66.8)	324 (46.1)	0.035*	0.24 (0.076)	0.24 (0.041)	0.932

PCF: pounds per cubic foot.

a Nonparametric Wilcoxon signed-rank test.

b Contains short glass fibre reinforcement.

* Statistically significant (p < 0.05).

### Perpendicular to foaming direction

**Figure 4.**
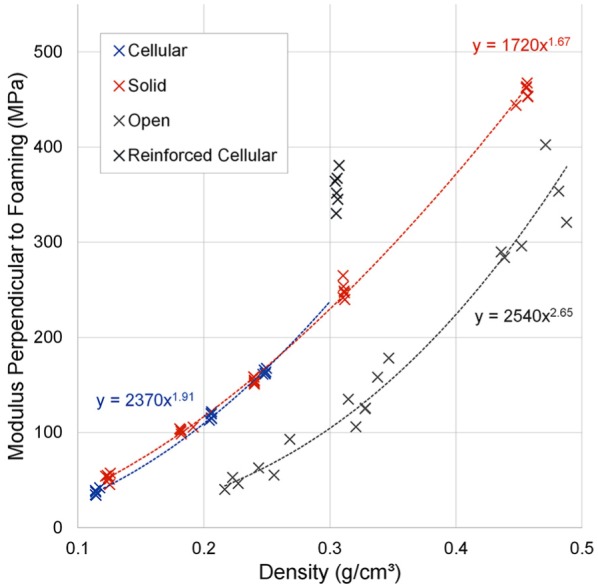
Summary of all point tracking results for tested foam types and densities compressed perpendicular to the foaming direction. 0.306 g/cm^3^ density cellular foam excluded from power law regression due to inclusion of fibre reinforcement.

A power law relationship was also observed (R^2^ > 0.98) between the measured Young’s modulus and density for all foam types when tested perpendicular to the foaming direction (Figure 4). Again, the 0.306 g/cm^3^ density cellular foam was an outlier to this trend. Using the power laws in the same way described above, the cellular foams had 2%–17% lower average Young’s modulus than solid foams, with the exception of the fibre-reinforced cellular 0.306 g/cm^3^ density foam, which was 43% higher. Open-cell foams had lower moduli on average; however, the differences were less pronounced (36%–72% of the solid foam modulus). All differences were significant (p < 0.005).

**Figure 5.**
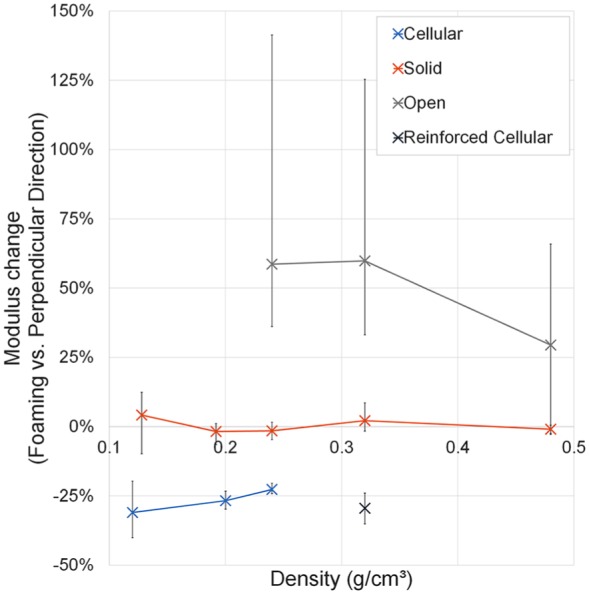
Percentage modulus differences between foams tested in the foaming and perpendicular directions for each foam type and density grouping. Error bars show range (min–max).

The measured Young’s modulus of cellular foam specimens compressed perpendicular to the foaming direction was on average 21%–31% lower than when compressed parallel to the foaming direction (p < 0.005, Table 3; Figure 5), with increased anisotropy at lower densities. Solid foam grades had no significant modulus differences between testing directions (p > 0.1). Open-celled foams had the reverse relationship to cellular foams, with Young’s modulus on average 29%–59% higher perpendicular to the foaming direction than parallel to it (p < 0.05).

### Poisson’s ratio

Poisson’s ratios of cellular foams were consistently higher when tested in the foaming direction (Table 3), decreasing with increasing density. As solid foams were more isotropic, Poisson’s ratios were consistent between testing directions and densities. Open-cell foams had a higher Poisson’s ratio in the foaming direction, converging to that of the transverse direction at higher densities.

## Discussion and conclusions

Young’s modulus was found to fit a power law in relation with density for each foam type tested, in agreement with trends found in literature.^8,12^ The outlier to this trend was the 0.306 g/cm^3^ density cellular foam, owing to its E-glass reinforcement. The inclusion of glass reinforcement has been reported to cause similar increases in compressive modulus for both PU^17^ and epoxy foams.^18^ The power law exponent of both solid and cellular foams was between 1 and 2, indicating a mixture of bending- and stretch-dominated loading behaviour, typically observed in closed-cell foams.^8^ The exponent of the open-cell foam was larger than two, indicating bending-dominated deformation consistent with open-cell foams.^8^

**Figure 6.**
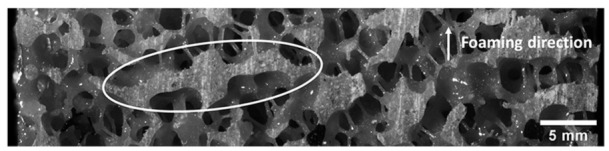
Example of solid material plane present in some open-cell foam specimens.

Of the foam types tested, both the cellular and open-cell foams were observed to have significantly different Young’s moduli between loading directions. The modulus of cellular foams was higher in the foaming direction, indicating elongation of cells in this direction. For the non-reinforced cellular foams, this effect was slightly reduced at higher densities, where pore sizes tend to be smaller.^12,19^ This implies that larger pore sizes promoted increased elongation, which has previously been observed by Gong et al.^20^ Young’s modulus of open-cell foams was found to be higher when tested perpendicular to the foaming direction than parallel to it. This is thought to result from solid planes of material present in specimens perpendicular to the foaming direction (Figure 6), which may have acted to stiffen the bulk material.

Cancellous bone displays considerable Young’s modulus anisotropy, varying with location as a result of preferred trabecular orientation dictated by Wolff’s law. The analogue bone materials in this study demonstrated anisotropy ratios consistent with reported values^21^ for real bone obtained from calcaneus and proximal femur locations, but lower anisotropy than observed from spinal and distal femur extraction sites.

Young’s modulus and Poisson’s ratio variation of cellular and solid foam types were low within batches. However, tested open-cell samples had considerable density, modulus and Poisson’s ratio variation. Inter- and intra-sample consistency was higher for the analogues than for real bone,^21^ except for the most dense tested open-cell foam. The larger Poisson’s ratio of cellular foams compressed in the foaming direction is thought to result from cell elongation effects as longer cells are more flexible laterally. Poisson’s ratio of solid foams was consistent between testing directions and densities as solid foams were more isotropic. Open-cell foams tended to exhibit a strain gradient as a result of higher constraint at one end.

The only study to the authors’ knowledge that measured Poisson’s ratio of biomechanical analogue foam materials was by Kelly and McGarry.^22^ They tested 8 mm side length Sawbone specimens with a 320 kg/m^3^ nominal density, cellular foam cubes, measuring transverse strain by video extensometry. They found Poisson’s ratio values between 0.14 and 0.28, generally lower than those found in this study. This may result from the small specimen dimensions tested in their study, in which test machine platen friction effects would have constrained transverse deformation across a larger proportion of the specimen.

Intra-sample variability of cellular and solid foams was low and similar in both testing directions. The intra-sample variability was considerably higher for open-cell foams. From Figures 3 and 4 it is apparent that most variation could be attributed to density differences. However, some variability is likely to result from the heterogeneous structure and solid planes of material.

This study made the assumption of transverse isotropy by testing Young’s modulus in one plane perpendicular to the foaming direction only. Intra-sample specimens were machined from single blocks of material, so further variability might be observed between blocks and batches. Cubic specimens could not be produced with the ASTM D1621 – 10 specified 51 mm side length as only 40-mm-thick blocks could be obtained. The non-contact optical strain estimation technique should minimise non-uniform loading and platen effects, but as a further mitigation measure, the larger 51 mm dimension was maintained for the non-foaming direction dimensions. Furthermore, bone may experience more complex loading including bending, tension and torsion, generating shear. The remit of this investigation was confined to compression, as the most common loading experienced by cancellous structures, and future work might characterise these materials’ shear moduli.

In conclusion, Young’s modulus and Poisson’s ratio of a range of commercially available analogue bone materials was characterised in compression both parallel and perpendicular to the foaming direction of production. Both the cellular and open-celled foam types showed significant modulus changes between testing directions while solid foams did not. As such, the anisotropic properties of analogue bones should be carefully considered when selecting an appropriate analogue testing material. For example, if researchers are trying to represent a relatively isotropic anatomic site, the solid foam material is appropriate. The open and cellular materials may be exploited to match the anisotropy of another anatomic site. Significant differences have been found between manufacturer-quoted and experimentally obtained Young’s modulus values for nominally the same material, dependent on the testing technique employed,^15,16^ and often the orientation of testing (perpendicular or parallel to the foaming direction) is not specified. Understanding these discrepancies becomes particularly relevant where the materials are used in standards testing and pre-clinical analysis of medical devices. If these materials are used in implantation studies involving press-fit fixation without careful knowledge of the material’s directional properties, corresponding uncertainty might be expected in the test results, such as the implantation force, implant and analogue deformations, and implant–analogue interface stability. Likewise, computational simulations employing these materials demand accurate input data, which can be acquired using a test technique which minimises experimental artefacts. This work provides researchers with a database of values to this end.
